# Comparison of depression level and identity styles between students in Allameh University and Islamic Seminary

**Published:** 2015

**Authors:** A Mahdavi, M Aghaei, MA Besharat, F Khaki Seddigh, SH Akbari, Z Hamidifar

**Affiliations:** * Clinical Psychology, Department of Psychology, Faculty of Psychology and Educational Sciences, University of Payame Noor, Tehran, Iran; ** General Psychology, Psychology Department, Faculty of Psychology and Educational Sciences, University of Tehran, Tehran, Iran; *** Clinical Psychology, Psychology Department, Faculty of Psychology and Educational Sciences, University of Tehran, Tehran, Iran; **** Department of Psychology, Faculty of Psychology and Educational Sciences, Islamic Azad University, Karaj Branch, Iran; ***** Rehabilitation Management, University of Social Welfare and Rehabilitation Sciences, Tehran, Iran; ****** General Psychology, Psychology Department, University of Payam Noor, Tehran, Iran

**Keywords:** depression, identity styles, students in Allameh University, students in Islamic Seminary

## Abstract

This research was conducted to compare the depression level and the identity styles between students in Allameh University and Islamic Seminary in Tehran city. The research method was the ex post facto or causal-comparative kind. In this research, all the students of Allameh University and Islamic Seminary were chosen as the research population. Among the statistical population, by using the convenience sampling method, a sample consisting of 100 male students was chosen (50-50 from both universities). Afterwards, the Identity Styles Inventory (ISI-6G) and the Beck Depression Inventory (21 questions) were employed in order to collect the data. By using ANOVA and systematic regression, the collected data were analyzed. The findings of the research indicated that the average values of the normative component (p-value = 0.03) and the depression level (p-value = 0.000) of seminary’s students were higher compared to the ones specific for the Allameh’s students. Among the various identity styles, commitment style could totally predict 16% of depression variable changes of Allameh’s students. Moreover, information and normative styles could totally predict 19% of the depression variable changes of the seminary’s students.

## Introduction

Depression is one of the most common psychiatric disorders that it is also referred to as psychological cold and chronically and in a relapsing manner, it decreases the educational, personal, and occupational function. The incidence during the lifetime is of 15%, and, in women, it reaches up to 25% [**1**]. According to Somsza E, Steer R, Beck AT, Clark DA (1994) [**2**], the depression symptoms are the following: general distress or negative emotion which is accompanied by general heartache, feeling guilty, fear, disturbance and hatred symptoms and also some special symptoms like depression due to the lack of positive emotion, i.e. disability in enjoying, sadness, anguish and disappointment. The data and self-related issues processing methodology could be based on variables affecting the mental health of individuals. In other words, frequency variables are correlated with disability to face mental pressure and among them identity could be mentioned [**3**]. Identity is defined as a coherent and stable feeling of who am I and who am I supposed to be [**4**]. Although the concept of identity was presented by Erickson, it was specified and presented in an original format by Marcia [**5**]. Marcia JE (1966) [**6**] introduced the identity as being a combination of beliefs, values, roles and behaviors and various cognitive, moral and practical skills which can be converted to its combined and coherent form, making the individual feel continuous about his/ her past and also projecting his/ her tendency related to the future. Marcia JE (1966) [**6**] specified multiple patterns based on Erickson’s identity concept i.e. “identity versus part confusion” by using a semi-organized interview, in which the subject between the ages of 18 and 25 is identified by the effort of youth. These four identity styles are identity gain, interval, identity record and identity dispersion [**6**]. The individuals who have a dispersed identity present no specified direction, do not adhere to goals and values, and do not bother to reach them. The individuals who have a recorded identity, have obligated themselves to the goals and values without assessing other options. The individuals who have an interval identity, did not create any definite obligation for themselves, looking for process, so that they were able to collect information and test various activities and hope that someday they will find some values and goals to direct their lives to. Finally, the individuals who have a gained identity have already assessed the options and they adhered to a series of values and goals that they have chosen. They have a mental health and the same feelings during time and they know where they are going [**6**]. According to Cock and Berzonsky (2005), male students had healthier lifestyle while female students reported more intimate interpersonal relationship and more tolerance against each other’s beliefs compared to male students. The results indicated that students with information style reported higher scores in educational autonomy, self-sufficiency, life effective management skills, respect and various individuals’ tolerance of themselves, creating intimate relationships, emotion independency, and self-confidence compared to students with confusion and normative identity styles. Also, students with a normative and information identity showed higher scores in the educational goals, realism, long-term plans about life and goals and stable relationships with peers compared to the students with a confused identity style. Moreover, the results of the above study indicated that informed students are healthier and have a better life style compared to the confused students. The results of Vleioras G, Bosma H (2005) [**7**] indicated that the avoidance of confronting the subjects related to identity has a negative relationship with psychological welfare, while when an individual or individuals confront(s) with identity subjects, their preferred way of facing with these subjects seems unimportant; in other words, there is no significant difference among individuals with normative and information identity in the psychological welfare scale. Based on the information mentioned above, the researcher seeks to compare depression and identity styles to specify in which of these groups the level of depression is higher, in addition, which of the identity styles is more prevalent in both groups, thus predicting the depression level.

## Methodology

The methodology was based on ex post facto or causal-comparative method. In this research, all the students in Allameh University and Islamic Seminary were chosen as a research population. Among the statistical population, a sample size including 100 male students (50-50 from both Allameh and seminary) were chosen by the convenience sampling method. After the attendance of the participants in an appropriate place and the coordination with the authorities and the reduction of the participants’ sensitivity to the questionnaires, explaining why they were selected in addition to gaining the required licenses, specifying sample members and coordinating with the related authorities, based on previous schedules, the required explanations were presented by the researcher regarding the questionnaires and the reasons of adding them to the sample size. Afterwards, they completed the questionnaires. Participants were asked to declare if they faced any ambiguity during completing the questionnaires, by just asking the researcher to provide more explanations. At the end, participants were appreciated for their cooperation. Then, the collected data were analyzed by using ANOVA and Regression statistical methods by employment of SPSS statistical software. The employed tools in the research were the following: 

**Identity Styles Inventory (ISI-6G)**

This inventory was initially designed by Berzonsky MD (1989) [**8**] to measure the social-cognitive processes that teenagers use in case of facing issues related to identity. According to Berzonsky, teenagers choose 4 different orientations or 4 different identity styles. This inventory evaluated four identity styles including information, normative, confusion/ avoidance and identity commitment that included 40 questions. The answer to each question was specified based on a 5-degree spectrum (totally disagree = 1, disagree = 2, nearly agree = 3, agree = 4, totally agree = 5). Considering the research conducted by Shokri O, Tajik Esmailie A, Daneshpour Z, Ghanaie Z, Dastjerdi R (2007) [**9**], the Cronbach’s alpha coefficients for information, normative, confusion/ avoidance and identity commitment were derived as 0.71, 0.53, 0.65 and 0. 72, respectively.

**Beck Depression Inventory (21 questions)**

This inventory was developed by Beck et al. in 1961. This 21-item self-report inventory was used to measure the depression’s severity and to determine the syndrome in normal individuals and patients. The inventory’s total validity that was conducted to evaluate the psychometric coordinates of this inventory in Iran was equal to 0.91 [**10**]. According to the research conducted on 196 male students in Shahid Chamran University, the Cronbach’s alpha coefficient for the whole inventory obtained was equal to 0.87. Split-half reliability coefficients and retest coefficient were reported as 0.89 and 0.49, respectively, with an interval of three weeks. Moreover, the correlation coefficient between the inventory and the depression subscales of MMPI questionnaire was calculated as 0.60 [**11**].

**Findings**

**Table 1:** Comparison between the means of seminary and Allameh’ students regarding other variables

Variable		Average	Standard deviation	Average of differences	Standard deviation of differences	Confidence interval of differences		t-statistics	Df	Significance level
						High level	Low level			
Normative	Allameh	38.32	6.30							
	Seminary	40.82	5.23	-2.54	1.15	-0.23	-4.84	-2.19	98	0.03
Information	Allameh	31.98	6.25							
	Seminary	32.38	5.69	-0.40	1.19	1.97	-2.77	-0.33	98	0.73
Avoidance	Allameh	26.28	5.70							
	Seminary	26.22	8.28	0.06	1.42	2.88	-2.76	0.04	98	0.96
Commitment	Allameh	35.34	5.32							
		37.52	6.88	-2.18	1.23	0.26	-4.62	-1.77	98	0.08
Depression	Allameh	14.38	7.59	-7.08	1.34	-4.40	-9.75	-5.24	98	0.000

Based on the table above, it could be deduced with a 95% certainty that there was a significant difference between the students in Allameh University and the ones in the Islamic seminary in terms of being normative (p-value = 0.03). In other words, the average value of being normative among the students in Islamic Seminary (40.82) was higher than in students in Allameh University (38.28). In addition, there was a significant difference between the mentioned students regarding the depression level (p-value = 0.000). In other words, by looking at the average values of depression in the two groups of students, it could be noted that the level of depression among the students in the Islamic Seminary (21.46) was higher than the one of the students in Allameh University (14.38). 

However, there was not any significant statistical difference between the mentioned students in terms of information, avoidance, and commitment at the 0.05 error level. 

**Multiple regression between depression and other factors among Allameh’s students**

H0: other variables together had no relationship with the depression of Allameh’s students.

H1: other variables together had no relationship with the depression of Allameh’s students. 

**Table 2:** Coefficient of model determination – Allameh’s students

Model	Correlation coefficient (r)	Determination coefficient (R2)
1	0.40	0.16

**Table 3:** Regression coefficients – Allameh’s students

Model	Variables	Variable coefficient(β)	t-Statistic (T)	Level of significance (p)
1	β0 – constant value	34.72	5.15	0.000
	X1 – being committed	- 0.57	- 3.05	0.004

The impact of the other independent variables (being normative, being informative, being avoidant and being committed) on the dependent variable (depression of Allameh’s students) was evaluated by using the systematic regression method and, as a result, one model was built. In this regression method, the variable of being committed was solely included in the model (P = 0.004, T = - 3.05). Based on Fisher’s test and the significance level of this model (P-Value = 0.004, F= 9.307), it could be expressed that the model had an acceptable validity. Moreover, the determination coefficient of the regression model was equal to the variable of being committed (R2 = 0.16), which showed the fact that the variable (being committed) could totally predict 16% of the changes of the dependent variable (depression of Allameh’s students). The regression line equation with a 0.05 error probability was the following:

Y= β0 + β1x1 + e

Depression of Allameh’s students = 34.72 – 0.57 (being committed) 

After fitting the model and predicting the depression values of Allameh’s students by using the multiple linear regression model, the evaluation of the normality of the data was started, which, for no reason, was achieved to reject the normality based on Kolmogorov-Smirnov test and its significance level (Kolmogorov-Smirnov Z = 0.781, p-value = 0.576).

**Multiple regression between depression and other factors among seminary’s students**

H0: other variables together had no relationship with the depression of seminary’s students.

H1: other variables together had no relationship with the depression of seminary’s students.

**Table 4:** Coefficient of model determination – seminary’s students

Model	Correlation coefficient (r)	Determination coefficient (R2)
1	0.28	0.08
2	0.43	0.19

**Table 5:** Regression coefficients – seminary’s students

Model	Variables	Variable coefficient(β)	Statistic (T)	Level of significance (p)
1	β0 – constant value	30.78	6.68	0.000
	X1 – being committed	- 0.29	- 2.05	0.045
2	β0 – constant value	20.71	3.44	0.001
	X1 – being committed	- 0.56	- 3.22	0.002
	X2 – being normative	0.46	2.45	0.018

The impact of other independent variables (being normative, being informative, being avoidant and being committed) on the dependent variable (depression of Allameh’s students) was evaluated by using the systematic regression method and two models were developed. As it was shown in the row related to model 1 of **Table 4**, the model that included only the information variable and constant value justified only 0.08 of the depression level dispersion of the seminary’s students. The value of determination coefficient reached up to 0.19 when the normative variable was added to model. Based on Fisher’s test and significance level of this model (P-Value = 0.008, F = 5.34), it could be noted that the model had an acceptable level of validity. Two variables of being avoidant and committed were not revealed in any of the two models since adding them to the model did not change the R2 value enough. Furthermore, the determination coefficient of the regression model was equal to the variables of being informative and normative (R2 = 0.19), which showed the fact that the variables (being informative and normative) could totally predict 19% of the changes of the dependent variable (depression of seminary’s students). The regression line equation with a 0.05 error probability is the following:

Y= β0 + β1x1 +β2x2 + e

Depression of seminary’s students = 20.71 – 0.56 (being informative) + 0.46 (being normative)

After fitting the model and predicting the depression values of the seminary’s students by using the multiple linear regression model, the evaluation of the normality of the data was started, which, for no reason was achieved to reject the normality based on Kolmogorov-Smirnov test and its significance level (Kolmogorov-Smirnov Z = 0.416, p-value = 0.995).

## Conclusion

The findings of the research indicated that there was no significant difference between the students in Allameh University and the ones of the Islamic seminary in terms of depression level and normative style. On the other hand, there was not any significant difference at the error level of 0.05 in terms of information, avoidance, and commitment styles. The impact of the identity styles on the depression level by using the systematic regression analysis method showed that the determination coefficient of the regression model was equal to the variable of being committed (R2 = 0.16), which represented the fact that the variable (being committed) could totally predict 16% of the changes in the depression variable among the Allameh’s students. In addition, the impact of identity styles on the depression level by using the systematic regression analysis method showed that the determination coefficient of the regression model was equal to the variables of being informative and normative (R2 = 0.19), which represented the fact that the variables (informative and normative) could totally predict 19% of the changes in the depression variable among the seminary’s students. The findings of this research could be regarded as a pattern in detecting the effective factors of depression. Furthermore, since identity styles are somehow formed in childhood and adolescence in a manner that their effects are revealed in adulthood, the results of this research could be used as prevention and educational procedures for parents, teachers and authorities to prevent depression. In fact, the results of this research were focused on the importance of designing a model that was based on training the identity styles and the methods of achieving a successful identity in adolescence and childhood.
